# Hyponatremia and malnutrition: a comprehensive review

**DOI:** 10.1007/s11845-023-03490-8

**Published:** 2023-09-13

**Authors:** German Baez, Martin Chirio, Pedro Pisula, Enrique Seminario, Natalia Carasa, Romina Philippi, Gustavo Aroca-Martinez, Carlos G. Musso

**Affiliations:** 1 Physiology Department, Instituto Universitario del Hospital Italiano de Buenos Aires, Buenos Aires, Argentina; 2 Nephrology Division, Hospital Italiano de Buenos Aires, Buenos Aires, Argentina; 3 Facultad de Ciencias de la Salud, Universidad Simón Bolívar, Carrera 59 No. 59-65, Barranquilla, Colombia

**Keywords:** Hyponatremia, Malnutrition, Pathophysiology

## Abstract

**Background:**

Hyponatremia (serum sodium lower than 135 mmol/L) is the most frequent electrolyte alteration diagnosed in medical practice. It has deleterious clinical effects, being an independent predictor of mortality. Malnutrition encompasses pathological states caused by both nutrients excess and deficiency, being frequently documented in chronic kidney disease patients. In addition, chronic hyponatremia promotes adiposity loss and sarcopenia, while malnutrition can induce hyponatremia. This pathological interaction is mediated by four main mechanisms: altered electrolyte body composition (low sodium, low potassium, low phosphorus, or high-water body content), systemic inflammation (cytokines increase), hormonal mechanisms (renin–angiotensin–aldosterone system activation, vasopressin release), and anorexia (primary or secondary).

**Conclusion:**

Malnutrition can induce hyponatremia through hydro-electrolytic, hormonal, inflammatory, or nutritional behavior changes; while hyponatremia per se can induce malnutrition, so there is a pathophysiological feedback between both conditions.

## Introduction

Hyponatremia, defined as natremia < 135 mmol/L, is the most frequent electrolyte alteration diagnosed in medical practice. Its importance relies not only on its deleterious effects, (neurologic symptoms, risk to falls, and osteoporosis development) but also on being an independent predictor of mortality. Probably, hyponatremia is simultaneously a severity marker of the underlying condition, and a direct contributor to poor prognosis [1].

The term *malnutrition* encompasses pathological states caused by both excess and deficiency of nutrients, which consists of a disorder of body composition characterized by an excess of extracellular water, frequently associated with a decrease in muscle and fat tissue, hypoproteinemia, and potassium deficiency. Moreover, this condition interferes with the host normal response to disease and treatment. Since malnutrition is frequently documented in chronic kidney disease (CKD) and end-stage renal disease (ESRD), the understanding of its pathophysiological mechanisms has led the International Society of Renal Nutrition and Metabolism (ISRNM) to coin a particular term to this condition known as *protein-energy wasting syndrome* (PEW) [2]. PEW is characterized by simultaneous loss of systemic body protein and energy storages, including muscle and fat wasting and visceral protein pool contraction. This phenomenon has been attributed to proinflammatory cytokines activation combined with superimposed hypercatabolic states, and appetite decline. The latter might be caused by uremia, or inflammation secondary to CKD inducing systemic conditions, such as diabetes mellitus or autoimmune diseases [3, 4]. The evidence tends to suggest that PEW develops more in patients from CKD stage 3b (estimated glomerular filtration rate < 45 mL/min) onwards, as defined by the Kidney Disease Improving Global Outcomes (KDIGO) staging of CKD [3].

In addition, it has been documented that PEW prevalence showed is 60–82% in AKI patients, 11–54% in 3–5 CKD patients, 28–52% in hemodialysis patients, and 40–45% in transplanted patients [4, 5]. Finally, malnutrition can be associated with *sarcopenia* and *cachexia.* Sarcopenia consists of significant muscle mass and strength loss of multifactorial etiology, which is associated with PEW in CKD patients, limiting patient’s autonomy and quality of life [6, 7]. Regarding cachexia, it refers to a very severe form of PEW, often associated with profound physiological, metabolic, psychological, and immunological disorders [4].

Hyponatremia and malnutrition are frequently found associated, and they have been even reported as associated with higher mortality, particularly in chronic hemodialysis patients [7–9]. In the present article, the fundamentals of the association between these two conditions are analyzed.

## Hyponatremia in malnutrition: its pathophysiology

1. Altered electrolyte body composition
  Five main hyponatremia inducing mechanisms have been, all of which can be documented in malnourished patients (Fig. 1):

**Fig. 1:**
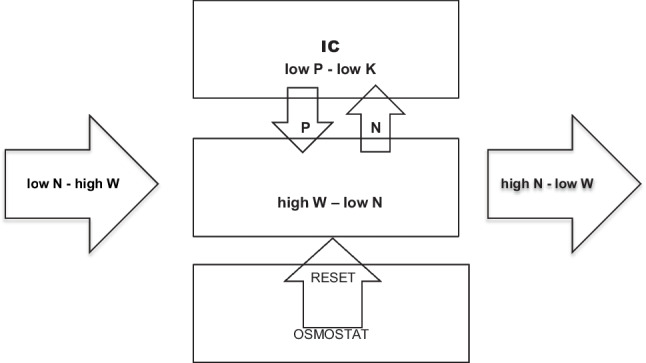
Pathophysiologic hyponatremia inducing mechanisms in malnourished patients. **IC**: intracellular compartment, **IV**: intravascular compartment, **N**: sodium, **W**: water, **P**: phosphorus, K: potassium
  Firstly, the mechanisms which consist of an inadequate salt and water excretion, which can be subdivided in three main categories: (a) depletion in excess of salt (e.g., chronic diarrhea with water replacement in malabsoption syndrome), (b) salt and water body retention in excess of water (with or without edema), which may present with hypervolemia (e.g., renal failure, etc.), or effective hypovolemia (cardiac failure, etc.) in inflammatory states associated with chronic disease, and (c) inadequate water retention, as it happens for instance in paraneoplastic antidiuresis syndromes [1, 7–9].
  Secondly, the mechanisms which consist of an inadequate salt and water income, which can be subdivided in two main categories: (a) low sodium diet, particularly in those malnourished patients suffering from anorexia and (b) excessive water income due to an increased thirst induced by high angiotensin II or inflammatory cytokines serum levels [9–14].
  Thirdly, the other factor which can modify the sodium/water ratio of the body is the body potassium content since its intracellular depletion leads to low serum sodium levels by inducing sodium shift to the intracellular compartment, as well as inappropriate vasopressin release [8–10]. Precisely, hyponatremia secondary to low potassium body content can be documented in severe malnourished patients. Moreover, sarcopenia is a condition usually documented in this population, and since muscle mass is the main potassium body store, sarcopenia represents reduced body potassium content. Therefore, it has been hypothesized that sarcopenia per se, as a cause of low potassium body content, could cause hyponatremia [10, 18].
  Edelman equation, a simplified version of Boling equation, summarized all these concepts in the following formula that describes how body sodium, potassium, and water determine serum sodium concentration [1]:
  $$\mathrm{Serum}\;\mathrm{sodium}\;\mathrm{concentration}\:=\:\frac{\mathrm{body}\;\mathrm{sodium}+\mathrm{body}\;\mathrm{potassium}}{\mathrm{total}\;\mathrm{body}\;\mathrm{water}}$$
  Fourthly, Zeballos et al. have postulated the reduced body phosphorus content usually found in malnourished peritoneal dialysis (PD) patients as an alternative cause of hyponatremia in this population. They hypothesized that in some PD patients during catabolic state, ribonucleic acid can break down, passing organic phosphates to inorganic phosphates. These anions would then leave the cells together with potassium in order to maintain electroneutrality, generating an osmotic effect that would dilute the intravascular compartment, favoring the appearance of hyponatremia [6, 19, 20].
  In the fifth place, reset osmostat (RO), a condition that has a low-normal plasma osmolality threshold which consequently induces vasopressin release at a lower plasma osmolarity, with normal water load excretion, and intact urine diluting ability, while maintaining normal sodium balance. RO has been documented in severely frail individuals [7–12].
  Sixth, there is the combination of some of the abovementioned mechanisms, which is usually documented in this population, as is the typical case of a patient suffering from chronic diarrhea (high sodium loss) and hyporexia (low sodium income).
  Seventh, it has already been documented a positive association between decreased lean body mass and low serum sodium levels, as well as that serum sodium tends to be low in the malnourished individuals [7, 8, 13, 14]. In this sense, Barsoney et al. have hypothesized based on animal studies that chronic hyponatremia can increase oxidative stress, promoting senescence manifestations, such as loss of adiposity, bone mass, and sarcopenia.
  Finally, malnutrition-associated chronic conditions (advanced heart failure, cirrhosis, AIDS, etc.) or malnutrition itself, through its inflammatory status, could also induce hyponatremia [11, 15–17].
  Since systemic inflammation, renin–angiotensin–aldosterone system (RAAS) activation, and anorexia are the main scenarios where the above-described hyponatremia inducing mechanisms are unfolded in malnourished individuals, they are described in detail as follows.
2. Systemic inflammation
  Inflammation is a possible pathogenetic pathway for the development of PEW and hyponatremia. On the one hand, chronic inflammation sends inflammatory signals to the hypothalamus which mediate energy wasting in chronic diseases. In addition, inflammatory activation of the hypothalamic receptors that control thirst has been suggested as another cause of hyponatremia by inducing excessive water ingestion [6, 8, 13, 21]. On the other hand, hyponatremia commonly appears in inflammatory states. This phenomenon could be explained since an interaction between interleukin-6 and vasopressin-induced antidiuresis has been proposed, based on animal experimental data [6, 8, 13, 22].
  Moreover, inflammation was associated with hyponatremia, independent from the presence or the absence of malnutrition. It has been observed an inverse relation between white blood cell count and serum sodium levels in hemodialysis patients, and hyponatremia was frequently associated with chronic infection, and other chronic inflammatory diseases. It has been suggested that either hyponatremia per se or by mucosal barrier breakdown through cellular edema could stimulate inflammation. Additionally, non-osmotic storage of sodium may induce the synthesis of interleukin-17 by CD4 + T-helper cells, which may contribute to chronic systemic inflammation [7, 8].
3. Hormonal mechanisms
  RASS can be activated by real or effective hypovolemia. Regarding the former, this can be documented in malnourished patients who suffer from volume contraction due to a chronic low sodium diet (salt restriction) and/or sustained sodium loss induced by undeclared diuretics or cathartic drugs intake (anorexia nervosa). Regarding the latter, this can be induced by low serum oncosis due to significant hypoalbuminemia (serum albumin ≤ 2 mg/L) in malnourished patients or it could be secondary to advanced edematous states (heart failure, cirrhosis, etc.) which have already developed malnutrition [1, 19].
  It has been documented that angiotensin II is a common mediator of muscle wasting and hyponatremia. Regarding the former, angiotensin II receptor type 1 is expressed in skeletal muscle and regulates its function, whereas elevated angiotensin II levels have been implicated in skeletal muscle atrophy. Regarding the latter, it has been described that elevated angiotensin II levels can be associated with polydipsia, and consequently, it can induce hyponatremia in some particular clinical settings (chronic kidney disease, etc.). Finally, inflammation-induced hyponatremia, mediated by inadequate vasopressin release, could also be a potential mechanism since angiotensin II is a proinflammatory factor [1].

### Anorexia

Anorexia of any origin is one of the hyponatremia inducing mechanisms in malnourished patients. This mechanism mainly induces hyponatremia by altering body electrolyte composition, as explained above. In a particular sort of anorexia, the anorexia nervosa [14, 22], which is the most life-threatening of all psychiatric diseases, medical complications due to malnutrition and/or purging behaviors are the main cause of mortality in these patients. In this sense, they are more susceptible to sodium and water depletion due to diuretic and/or laxative abuse, and vomiting in a context of inadequate intake of fluids and sodium in defect of sodium (long-term sodium restriction). This nutritional behavior of not adding salt to food is usually used as a method to control body weight in this population. An inadequate vasopressin secretion can also contribute to induced hyponatremia in these patients [23].

## Conclusion

Malnutrition can induce hyponatremia through hydro-electrolytic, hormonal, inflammatory, or nutritional behavior changes; while hyponatremia per se can induce malnutrition, then there is a pathophysiological feedback between both conditions.
